# The impact of tubal ectopic pregnancy in Papua New Guinea – a retrospective case review

**DOI:** 10.1186/1471-2393-13-86

**Published:** 2013-04-04

**Authors:** Nancy N Hamura, John W Bolnga, Regina Wangnapi, Andrew W Horne, Stephen J Rogerson, Holger W Unger

**Affiliations:** 1 Department of Obstetrics and Gynaecology, Modilon General Hospital, P.O. Box 2119, Madang, 511, Papua New Guinea; 2 Papua New Guinea Institute of Medical Research, PO Box 378, Madang, 511, Papua New Guinea; 3 MRC Centre for Reproductive Health, The Queen’s Medical Research Institute, The University of Edinburgh, Edinburgh, UK; 4 Department of Medicine (Royal Melbourne Hospital), The University of Melbourne, Melbourne, Australia

**Keywords:** Delays, Ectopic pregnancy, Management, Papua New Guinea, Rate, Resource-limited settings

## Abstract

**Background:**

Ectopic pregnancy (EP) is an important cause of morbidity and mortality amongst women of reproductive age. Tubal EP is well described in industrialised countries, but less is known about its impact in low-resource countries, in particular in the South Pacific Region.

**Methods:**

We undertook a retrospective review of women with tubal EP treated at a provincial referral hospital in coastal Papua New Guinea over a period of 56 months. Demographic and clinical variables were obtained from patients’ medical records and analysed. The institutional rate of tubal EP was calculated, and diagnosis and management reviewed. Potential risk factors for tubal EP were identified, and delays contributing to increased morbidity described.

**Results:**

A total of 73 women had tubal EP. The institutional rate of tubal EP over the study period was 6.3 per 1,000 deliveries. There were no maternal deaths due to EP. The mean age of women was 31.5+/−5.7 years, 85% were parous, 67% were rural dwellers and 62% had a history of sub-fertility. The most commonly used diagnostic aid was culdocentesis. One third of women had clinical evidence of shock on arrival. All women with tubal EP were managed by open salpingectomy. Tubal rupture was confirmed for 48% of patients and was more common amongst rural dwellers. Forty-three percent of women had macroscopic evidence of pelvic infection. Two-thirds of patients received blood transfusions, and post-operative recovery lasted six days on average. Late presentation, lack of clinical suspicion, and delays with receiving appropriate treatments were observed.

**Conclusions:**

Tubal EP is a common gynaecological emergency in a referral hospital in coastal PNG, and causes significant morbidity, in particular amongst women residing in rural areas. Sexually transmitted infections are likely to represent the most important risk factor for tubal EP in PNG. Interventions to reduce the morbidity due to tubal EP include the prevention, detection and treatment of sexually transmitted infections, identification and reduction of barriers to prompt presentation, increasing health workers’ awareness of ectopic pregnancy, providing pregnancy test kits to rural health centres, and strengthening hospital blood transfusion services, including facilities for autotransfusion.

## Background

Ectopic pregnancy (EP) is the implantation of a fertilised ovum outside the uterine cavity [1]. EP most commonly occurs in the fallopian tubes (> 98%) [2]. If left untreated, or diagnosed late, EP can result in intra-abdominal haemorrhage and death [3]. In high-income countries 1-2% of all pregnancies are EPs [2]. Their incidence has risen, and they frequently represent the leading cause of early maternal mortality [3,4]. Fallopian tube damage secondary to surgery or genital tract infection (in particular *Chlamydia trachomatis*), infertility and sub-fertility, contraceptive failure, increased maternal age, smoking, and a history of previous EP and miscarriage are well-established risk factors for tubal EP [2]. In well-resourced healthcare systems the wide availability of, and access to, ultrasound, urine and serum β-human chorionic gonadotrophin (β-HCG) tests, and diagnostic laparoscopy facilitates diagnosis [2]. Most tubal EPs are removed laparoscopically (provided the patient is haemodynamically stable), with an increasing number of unruptured tubal EPs treated medically [4].

Less is known about the burden and management of EP in low and middle-income countries (LMICs). Reports on the contribution of EP to maternal deaths provide conflicting data: one meta-analysis estimates it is < 1% [5], whereas other, mostly African, studies report it is higher than in high-income countries [6-10]. In Asia, mortality rates due to EP appear more homogenous and are lower compared to Africa [7,10,11]. Given the possible shortcomings in documentation and reporting, as well as the possibility of affected women not seeking or reaching healthcare facilities, a clear consensus on the incidence of EP and its relative contribution to maternal morbidity and mortality in LMICs is lacking. Equally, the use of different denominators for reporting EP rates hinders comparison internationally (e.g. number of EP per 1,000 deliveries, or per 10,000 women at reproductive age) [12]. Nevertheless, available data suggests EP is a relatively common gynaecological emergency in LMICs, its case fatality is high [7] and its incidence is rising [6,7,13].

Risk factors associated with tubal EP in LMICs are likely to be similar to those observed in industrialised countries, although relative contributions may differ and regional heterogeneity is likely to exist. Previous use of assisted reproductive technology and contraceptive failure are less commonly implicated in LMICs [9]. Chlamydial (and possibly *Neisseria gonorrhoeae*) infection and pelvic inflammatory disease may be more important [7,9,14-16], and so is smoking, given the increasing number of women at reproductive age in LMICs who use tobacco [17]. In addition, there are indications that tuberculosis and *Schistosoma haematobium* infection may increase the risk of tubal EP [18-20]. Behavioural and clinical risk factors include early sexual debut, multiple lifetime sexual partners, lack of condom use, miscarriage and induced abortion, and a history of pelvic inflammatory disease (PID) and sexually transmitted infection (STI) [9].

The most frequently used treatment modality for tubal EP in LMICs is open salpingectomy [6,21,22]: many tubal EPs have ruptured by the time women reach hospital and/or a diagnosis has been made [6,21,23-29]. Reasons for this may include women’s unawareness of being pregnant and underestimation of seriousness of symptoms, as well as late diagnosis or misdiagnosis by healthcare providers [21,30,31]. Assessments of delays with seeking and receiving appropriate care, as applied in maternal death reviews, are absent for women with tubal EP in LMICs [3,32].

Papua New Guinea (PNG) is a LMIC situated north of Australia with approximately seven million inhabitants of Melanesian background [33]. Recent surveys confirm a high fertility rate of 4.1-4.4 births per woman [34,35] and maternal death is common [35-37]. Up-to-date information on EP in PNG is limited [38-43]. Sanga et al. reported two maternal deaths due to tubal EP at a provincial referral hospital in the Highlands region, accounting for 6.8% of all maternal deaths observed during their study period (2005–2008) [38]. In the wider South Pacific region, only one report from Vanuatu on incidence and management of tubal EP in a population of largely Melanesian background exists, which is 20 years old [23].

This study aimed to evaluate the incidence, diagnosis and clinical management of tubal ectopic pregnancy in a provincial referral hospital in PNG.

## Methods

### Study design

This was a retrospective review of women with tubal EP managed at Modilon General Hospital (MGH), Madang Province, PNG, during the period 01/01/2008 – 31/08/2012. The operating theatre register and gynaecology ward admission book were used to identify patients who had been booked for surgery for, or upon surgery were diagnosed with, tubal EP. The emergency case register was screened to identify potential tubal EP cases that were not managed surgically. Through cross-comparison with the hospital medical record registers additional cases were identified. Women’s charts were subsequently obtained and reviewed in detail.

### Data collection

Demographic and clinical variables were extracted from patients’ charts and entered into an Excel spreadsheet. Information collected included demographic variables such as age, ethnicity and area of residence, and data on clinical management including laboratory data. Each case was allocated a unique identification number, ensuring anonymisation in subsequent analysis. The labour ward register was used to collect information on deliveries during the study period.

### Data analysis

Data analysis was performed using Excel and STATA version 10.0 (StataCorp, College Station, USA). Institutional tubal EP rates were calculated per 1,000 births (live births and stillbirths ≥ 24 weeks’ gestation) [12]. Delays were categorised according to the ‘three phases of delay’ model [32]. Phase one delays were those with clear evidence of a delay with seeking care. Phase two delays included all women with delays in reaching the health centre and/or hospital due to transport problems or distance (including delays with transfers from health centres to MGH). Phase three delays included all women who had evidence of delays with receiving appropriate care in a timely fashion – delays of this category were analysed separately for health centres and hospital. Only delays that could be identified beyond doubt using all available documentation of individual patient journeys were included. Written ethical approval for this review was obtained from the MGH directorate.

## Results

A total of 91 possible tubal ectopic pregnancy cases were identified during the study period. Case notes were available for 81 women of whom 73 had tubal ectopic pregnancies confirmed clinically at surgery (including 2 heterotopic pregnancies with tubal implantation). In addition, there were three ovarian ectopic pregnancies, and one chronic abdominal pregnancy (lithopaedion). The institutional rate of tubal EP during the study period was 6.3 per 1,000 deliveries, which equals 1 tubal EP per 159 deliveries (Table 1). None of the 77 women with confirmed ectopic pregnancy (any type) died. Four cases were not EP: women underwent surgery for clinical suspicion of EP but were subsequently confirmed to have viable intrauterine pregnancies (n = 3) or ruptured luteal cysts (n = 1).

**Table 1:** Rates of tubal ectopic pregnancy at Modilon General Hospital during the period 01 Jan 2008 – 31 Aug 2012

	**2008**	**2009**	**2010**	**2011**	2012*	**Total**
Tubal ectopic pregnancies	11	15	20	20	7	73
Total deliveries**	1708	2113	2763	2842	2141	11567
Rate (tubal ectopics only)***	6.4	7.1	7.2	7.0	3.3	**6.3**

* 01/01/2012 – 31/08/2012 only; ** live births and stillbirths ≥ 24 weeks’ gestation; *** Number of ectopics/1,000 deliveries.

### Socio-demographic background of women with tubal ectopic pregnancy

The mean age of women with tubal EP was 31.5 +/− 5.7years (median = 31, range 15 – 45; n = 73) and the majority (95%; 69/73) were aged ≥ 25 years. Most women (93%; 62/67) were married, three were in *de facto* relationships and two were single. Forty-nine women (67%; 49/73) were rural dwellers. Fifty-six women (79%; 56/71) belonged to ethnicities home to Madang Province. Women’s employment and educational status was documented poorly.

### Clinical background and risk factors of patients with tubal EP

The median parity was 2 (IQR = 1–3, mode: 1; range: 0–8; n = 72): 11 (15%) women were nulliparous, 41 (56%) were P1-2, 10 (14%) P3-4, and 10 (14%) were ≥ P5. Documentation of data was insufficient to calculate gestational age at presentation. Forty-five women (62%) had evidence of sub-fertility (defined as last childbirth ≥ 3 years ago, or being nulliparous and having attempted to conceive for > 2 years). Ten patients had a documented history of PID of whom eight also had a history of sub-fertility, and three had a history of STI. Six subjects had a tubal EP previously (all managed through open salpingectomy), and five had suffered miscarriage before. None of the women reported prior use of an intrauterine contraceptive device. Smoking status was only documented for 24 women – four were smokers.

### Clinical findings and management of tubal ectopic pregnancy

The most common presenting complaint was abdominal pain, followed by abnormal vaginal bleeding, and one third of women had clinical evidence of shock (Table 2). Pre-operative haemoglobin (Hb) levels ranged from 3.6 – 15.0 g/dL (mean 8.5, median 8.0) (n = 60), and one third of patients had Hb levels of < 7 g/dL (n = 21). All 73 women with tubal EP underwent laparotomy under general anaesthesia. Most tubal EPs were managed by salpingectomy (n = 61/71); seven patients required a salpingo-oophorectomy and three had partial myomectomies for interstitial ectopics. Eleven patients simultaneously underwent ligation of the contralateral tube. There was one case of peri-operative bowel injury. All patients received peri- and postoperative broad-spectrum antibiotic cover as per national guideline [44]. Forty-nine patients (64%) received blood transfusions: the median number of units given was 2 (n = 32). Facilities for autotranfusion and rhesus blood group testing were not available at MGH during the study period.

**Table 2:** Common signs and symptoms observed amongst women with tubal ectopic pregnancy (n = 73)

**Investigation**	**% (number)**
*Presenting complaints*	
Abdominal pain	98.6 (72)
Abnormal vaginal bleeding	60.3 (44)
Amenorrhoea	52.1 (38)
Syncope*	26.0 (19)
*Findings upon examination*	
Guarding	56.2 (41)
Abdominal distension	43.8 (32)
Shock**	34.3 (25)
Rebound tenderness	32.9 (24)
Pyrexia	24.7 (18)
Pallor	23.3 (17)
Rigid abdomen	19.2 (14)
Abdominal mass	5.5 (4)
Bulging Pouch of Douglas	35.6 (26)
Cervical excitation	32.9 (24)
Adnexal tenderness	27.4 (20)
Adnexal mass	6.9 (5)

* Less common symptoms included nausea and vomiting, vaginal discharge, shoulder tip pain, dysuria, pain on defaecation and shortness of breath.

** Defined as heart rate > 90 beats per minute and blood pressure ≤ 90/60 mmHg.

Fourteen women had delays with discharge from hospital due to post-operative pyrexia (n = 11), anaesthetic complications (n = 2), and severe anaemia (n = 1). The mean number of days from surgery until discharge was 6 (median = 5, range 3 – 12; n = 70). For women with a remaining fallopian tube hormonal contraception was not prescribed, and information on subsequent conception success was unavailable.

### Operative findings

There were 39 left and 34 right tubal EPs. Information on tubal location was available for 58 women: 25 EPs were fimbrial, 24 were ampullary, 6 were isthmal and 3 were interstitial. Macroscopic evidence of pelvic infection, e.g. adhesions and hydrosalpinx, was noted in 31 women with tubal EP. Thirty-five women with tubal EPs (48%) had evidence of rupture. They were more likely to be rural dwellers (Fisher’s exact test, p = 0.023) compared to women with unruptured EPs, but there was no difference in parity (Mann–Whitney *U* test, p = 0.89) and age (unpaired *t*-test, p = 0.22) between groups. Intra-abdominal bleeding (any amount) was observed in 67 women (92%). When documented, estimated intra-abdominal blood loss volume (haematoperitoneum) ranged from 100 mls to 3000 mls (mean = 1331 mls, median 1500 mls; n = 64). Two women had evidence of endometriosis.

### Diagnosis and delays

Of 73 women with tubal EP, 34 (47%) were referrals from other health centres and aid posts. Amongst them, 33 (97%) patients had documentation of a differential diagnosis made at the referral centre: EP was considered in 14 cases (42%). There was no use of pregnancy tests at health centre level. Upon presentation to hospital, a differential diagnosis made by junior medical staff was documented for 47 (64%) patients: EP was considered for 31 of these women (66%). Investigations undertaken at MGH to aid diagnosis of tubal EP included culdocentesis (most frequently used), urinary β-HCG, and ultrasound (Table 3). Forty patients (55%) with tubal EP had evidence of a delay with seeking healthcare (phase one delay). Four patients had a phase two delay. Twenty-two and 27 patients experienced a delay with receiving appropriate care and treatment at referral centre and hospital, respectively. Twenty-nine patients had more than one delay, and 12 (16%) had no delay at all. Typical phase three delays at health centre level included patients not receiving necessary treatment as per national guidelines, e.g. intravenous fluids and antibiotics (n = 18) [44]. Misdiagnosis resulted in delays with referral to MGH for four patients (patients with suspected miscarriage were referred promptly). Phase three delays at hospital level included delays with appropriate management due to misdiagnosis (n = 14), delays with receiving prompt surgery (n = 8), delays with blood transfusion (n = 5), and delays with prompt medical review and intra-hospital referral (n = 2).

**Table 3:** Investigations performed to aid diagnosis of ectopic pregnancy (n = 81; all suspected cases with charts)

**Investigation**	**All suspected cases**	**Women with tubal ectopic pregnancy**	**Women with extra-tubular ectopic pregnancy**	**Women with no ectopic pregnancy**
	**(n = 81)**	**(n = 73)**	**(n = 4)**	**(n = 4)**
Culdocentesis; % (n)	88.9 (72/81)	89.0 (65/73)	75.0 (3/4)	100.0 (4/4)
*Positive;* % (n)	88.9 (64/72)	92.3 (60/65)	66.7 (2/3)	50.0 (2/4)
β-HCG*; % (n)	65.4 (53/81)	65.8 (48/73)	25.0 (1/4)	100.0 (4/4)
*Positive;* % (n)	94.3 (50/53)	95.8 (46/48)	100.0 (1/1)	75.0 (3/4)
Ultrasound**; % (n)	39.5 (32/81)	39.7 (29/73)	50.0 (2/4)	25.0 (1/4)
*Positive;* % (n)	93.8 (30/32)	96.6 (28/29)	100.0 (2/2)	0.0 (0/1)

* urinary human chorionic gonadotrophin ** extra-uterine gestational sac and/or free intra-abdominal fluid.

## Discussion

Ectopic pregnancy is a common gynaecological emergency at a provincial referral hospital in coastal PNG: we counted 6.3 tubal EPs per 1,000 deliveries at MGH. Although maternal deaths due to EP were not observed, morbidity was high: the majority of patients with tubal EP had significant intra-abdominal bleeding, half of them had evidence of tubal rupture, and one-third of patients were in hypovolaemic shock on arrival. All patients with tubal EP were managed by open salpingectomy, and two-thirds received blood transfusion peri- or postoperatively. Women residing in rural areas were more likely to have a ruptured tubal EP. This requires consideration at several levels.

The institutional rate of tubal EP provides an insight into the burden of EP in PNG, but cannot be used to determine overall regional incidence. It is likely that proportionally more ectopic pregnancies ‘occur’ at a referral hospital, and the overall regional rate may be lower. For instance, if only direct hospital presentations were included, the EP rate would halve. Furthermore, 16 of 37 of non-referrals were residing in distant rural areas. However, this may be offset in part by those EPs that may go undetected in rural areas, in particular in view of the low rate of clinical suspicion observed at health posts. By the time hospital was reached, many women were already suffering haemodynamic compromise: this raises concerns about women dying from EP without ever reaching a healthcare facility. EP has been observed to be a cause of early maternal death in other areas in PNG, and it is possible that its overall contribution to maternal deaths in PNG and other LMICs is underestimated [38]. In addition, case notes were unavailable for ten potential cases of EP. Our institutional rate is similar to most rates observed in LMICs in Asia [11,13], but lower compared to rates reported from Europe and Africa [9,21,31,45-48]. Underdetection of cases and differences in the frequency and distribution of risk factors for tubal EP are likely to be implicated.

Delays with presentation to a healthcare provider (health centre or hospital) were common, and may have contributed to the high proportion of women with tubal rupture. The distribution of phase one delays between rural and urban dwellers was similar (data not shown), and root causes for late presentation remain unclear. It is possible that women may be unaware of their pregnancy status and pregnancy-related complications, or indeed may hesitate to seek help as they find it difficult to raise funds for health centre visits. The number of clearly identifiable phase two delays in our study was low; however, rural dwellers were more likely to suffer tubal rupture. It is therefore possible that phase two delays were underestimated, due to intrinsic constraints of retrospective case note reviews. Phase three delays included problems with diagnosis: at health centre level pregnancy test kits were not used (possibly due to unavailability) which may have increased the time until a diagnosis was made and referral was arranged. At hospital level, diagnostic tools were more readily available and aided diagnosis in the majority of cases (Table 3).

The high proportion of women where EP was not suspected as part of the initial differential diagnosis is concerning. Interventions to increase health workers’ awareness of EP are of utmost importance. Delays with timely transfusion and prompt surgery were also noted, pointing towards the need to increase laboratory and operative capacity in referral hospitals. Autotransfusion could be one approach to reduce the number of donated units of blood required [29].

Culdocentesis is frequently used, and together with pregnancy test kits is likely to remain the main diagnostic tool to complement clinical history and examination in PNG in the short term. Ultrasound is gradually introduced but its application is limited by cost and lack of trained staff. In the longer term, research into identifying serum biomarkers for the rapid diagnosis of tubal EP is important. Such a biomarker could provide clinical staff working in low-resource environments with a simple rapid bedside diagnostic test, aiding prompt diagnosis and management and reducing morbidity and healthcare costs [49,50]. However, its potential success would still rely on clinical suspicion of EP and functioning supply chains ensuring adequate distribution, the latter yet unattainable for pregnancy test kits.

For most women in PNG, the clinical course of tubal EP would preclude medical management with methotrexate. Facilities for laparoscopic surgery were unavailable at MGH, but its introduction could aid diagnosis and reduce post-operative recovery time and morbidity. Nevertheless, at least one third of patients were in haemodynamic shock, and laparotomy was the operation of choice. This echoes findings from research conducted in other LMICs [13,20,45,47]. Equally, the role of salpingotomy may be limited – most women with EP were parous, tubal rupture was common, and tubal preservation may not be viewed as a major clinical concern in this patient population (yet).

There is evidence that the prevalence of STIs, including *C. trachomatis* and *N.gonorrhoeae*, is high in PNG [51,52]. In addition, in PNG early sexual debut and low condom use increase the risk of STIs [53]. Although only ten patients had a documented history of PID in this review, 31 (43%) had macroscopic evidence of PID at surgery and 62% (n = 45) of women with tubal EP had a history of subfertility. It is likely that PID represents an important risk factor for tubal EP in PNG and similar observations have been made in other LMIC environments [14,45]. Early sexual debut and low uptake of contraception may in part explain that the majority of women (85%) with tubal EP were parous. It remains unclear however, why the mean age of patients with tubal EP (31.5 years) is comparatively higher compared to other studies from LMICs [25,26,47,54-56]. Another potentially important risk factor may be tobacco smoking. In this study there were only four women who smoked; however, documentation of smoking habit was lacking for 67% of patients. Smoking may become an increasingly important risk factor for tubal EP in PNG given the fact that the number of women who smoke is rising [17]. Lastly, betel nut (*Areca catechu* L.) consumption amongst pregnant women in Madang Province is common and known to increase the risk of adverse pregnancy outcomes [57]. A possible association between this habit, tubal damage and an increased risk of EP has not been examined yet. Case notes used in this study did not contain information on betel nut consumption.

The PNG manual of standard managements in obstetrics and gynaecology is a highly useful resource for health workers in PNG [44], and it includes guidelines for the management of EP. Hormonal contraception for a minimum of six months is recommended for patients with a remaining tube following surgery. However, none of the relevant cases in this review were managed accordingly. Equally, when EP is suspected, fluid resuscitation prior to referral to hospital is advocated, but this was frequently not done. It is unclear as to whether this was due a lack of resources, or guidelines were not followed. Lastly, haematinic supplementation is recommended upon discharge, but was commonly not described (data not shown) [44]. Wide distribution of the manual, concomitant training and adequate medical supply chains are required to ensure optimal management of EP in PNG.

This review has identified areas of further research. A prospective institutional case–control study can assist with confirming demographic and clinical risk factors for tubal EP in PNG, and could be combined with STI screening. In addition, such study could examine the sensitivity and specificity of currently available diagnostic aids (namely culdocentesis and urinary β-HCG) in the PNG clinical environment: we identified four women with negative findings on laparotomy, yet two of them had a positive culdocentesis (Table 3). Such cases need to be reviewed when they occur to assess whether they could have been prevented through appropriate use of clinical history and examination and currently available diagnostic aids. Semi-structured interviews with women diagnosed with tubal EP will clarify the root causes of potential delays experienced (in particular phase one and phase three delays). The role of family and community care to improve diagnosis and prompt management of EP could be evaluated simultaneously [58]. Detailed post-mortem examinations of women who died at reproductive age are required to understand the contribution of EP to maternal mortality; however, facilities for this are currently unavailable at MGH, and this may not be applicable to women who died in distant rural communities. Here, qualitative research methodology may help identify EP as a potential cause of death. In addition, this study has shown that EP is frequently not suspected as a potential diagnosis – a survey on knowledge and awareness amongst health workers may identify needs for further training. Lastly, and perhaps most importantly, research into knowledge, beliefs, attitudes and practices relating to family planning, conception and early pregnancy amongst women of reproductive age needs to be conducted: it is possible that many are not aware of being pregnant [21], and delays with presentation increase the risk of tubal rupture, reduce the window of opportunity for medical management and enhance the risk of morbidity and mortality.

There are several limitations to the study. First, notes for ten potential (yet unconfirmed) cases were unavailable for review. This could result in underestimation of the institutional tubal EP rate. Second, the review was based on case note data alone, and is therefore subject to documentation bias. In particular, socio-economic patient background data was poorly documented. Third, documentation of the patient journey was not always detailed enough to detect a delay – only delays clearly identifiable from case notes were included and the true number of delays may be underestimated, in particular phase two delays. Furthermore, when delays were detected we were not always able to identify the root cause(s) of the delay. For instance, it was unclear why some patients did not receive antibiotics and/or intravenous fluids at some health centres: inadequate stocks, misdiagnosis and suboptimal management are all possible explanations. Lastly, it is possible that EPs causing maternal death may have been missed. One of the ten patients’ cases notes that were unavailable belonged to a young woman who collapsed on arrival at MGH and died – a differential diagnosis of ectopic pregnancy was provided, but could not be substantiated through documentation of a positive pregnancy test result or autopsy findings.

## Conclusions

Tubal EP is a common gynaecological emergency in a provincial referral hospital in coastal PNG, and causes significant maternal morbidity. Most women had evidence of intra-abdominal bleeding, and tubal rupture was common, in particular amongst patients from rural areas. Clinical suspicion of EP at aid posts was low, and simple diagnostic aids such as pregnancy test kits are underused or not available at these sites. Macroscopic evidence of pelvic infection was common and the prevalence of STIs in PNG is high [52] – this points towards STIs as the most important risk factor for tubal EP in PNG. In addition to preventing, detecting and treating STIs, interventions to reduce morbidity (and mortality) include family planning and the identification and reduction of barriers to early presentation. Increasing health workers’ knowledge of the condition, and providing them with simple clinical and diagnostic tools (in particular pregnancy test kits) will improve diagnosis and referral. At hospital level, prompt review and diagnosis of cases, and strengthening operating and transfusion capacities will reduce morbidity.

## Competing interest

Andrew Horne holds a UK patent for a diagnostic biomarker for ectopic pregnancy (# 0712801.0). All other authors declare no competing interest.

## Authors’ contributions

NH, JB and HWU initiated the review and collected and analysed the data. NH, HWU, JB, RW, AWH and SJR drafted and critically revised the manuscript. All authors read and approved the final manuscript.

## Pre-publication history

The pre-publication history for this paper can be accessed here:

http://www.biomedcentral.com/1471-2393/13/86/prepub

## References

[B1] Farquhar CM Ectopic pregnancy Lancet 2005 366 9485 583 591 10.1016/S0140-6736(05)67103-6 16099295

[B2] Sivalingam VN Duncan WC Kirk E Shephard LA Horne AW Diagnosis and management of ectopic pregnancy J Fam Plann Reprod Health Care 2011 37 4 231 240 10.1136/jfprhc-2011-0073 21727242 PMC3213855

[B3] Cantwell R Clutton-Brock T Cooper G Dawson A Drife J Garrod D Harper A Hulbert D Lucas S McClure J Saving Mothers’ lives: reviewing maternal deaths to make motherhood safer: 2006–2008. The eighth report of the confidential enquiries into maternal deaths in the United Kingdom BJOG 2011 118 Suppl 1 1 203 21356004 10.1111/j.1471-0528.2010.02847.x

[B4] Jurkovic D Wilkinson H Diagnosis and management of ectopic pregnancy BMJ 2011 342 d3397 10.1136/bmj.d3397 21665933

[B5] Khan KS Wojdyla D Say L Gulmezoglu AM Van Look PF WHO analysis of causes of maternal death: a systematic review Lancet 2006 367 9516 1066 1074 10.1016/S0140-6736(06)68397-9 16581405

[B6] Thonneau P Hijazi Y Goyaux N Calvez T Keita N Ectopic pregnancy in Conakry, Guinea Bull World Health Organ 2002 80 5 365 370 12077611 PMC2567787

[B7] Goyaux N Leke R Keita N Thonneau P Ectopic pregnancy in African developing countries Acta Obstet Gynecol Scand 2003 82 4 305 312 10.1034/j.1600-0412.2003.00175.x 12716313

[B8] Fubara DS Ikimalo J John CT Pathology of maternal deaths in Rivers state (a ten year autopsy review) in a referral hospital Niger Postgrad Med J 2007 14 3 256 260 17767214

[B9] Anorlu RI Oluwole A Abudu OO Adebajo S Risk factors for ectopic pregnancy in Lagos, Nigeria Acta Obstet Gynecol Scand 2005 84 2 184 188 15683381 10.1111/j.0001-6349.2005.00684.x

[B10] Indian Council of Medical Research Task Force Project Multicentre case–control study of ectopic pregnancy in India J Gynaecol Obstet India 1990 40 425 430

[B11] Ehsan N Mehmood A Ectopic pregnancy: an analysis of 62 cases J Pak Med Assoc 1998 48 2 26 29 9610087

[B12] de Rosnay P Irvine LM Reporting rates of ectopic pregnancy: are we any closer to achieving consensus? J Obstet Gynaecol 2012 32 1 64 67 10.3109/01443615.2011.618894 22185541

[B13] Bag TS Saha DP Dasgupta N Sarkar M Mandal SK Mondal T Saha SK Time trends in ectopic pregnancy over a decade–a retrospective hospital-based study J Indian Med Assoc 2012 109 10 727 729 22482318

[B14] De Muylder X Ectopic pregnancy in Zimbabwe Int J Gynaecol Obstet 1991 35 1 55 60 10.1016/0020-7292(91)90064-C 1680077

[B15] Ville Y Leruez M Glowaczower E Robertson JN Ward ME The role of Chlamydia trachomatis and Neisseria gonorrhoeae in the aetiology of ectopic pregnancy in Gabon Br J Obstet Gynaecol 1991 98 12 1260 1266 10.1111/j.1471-0528.1991.tb15399.x 1777459

[B16] Picaud A Berthonneau JP Nlome-Nze AR Ogowet-Igumu N Engongah-Beka T Faye A Serology of Chlamydia and ectopic pregnancies. Incidence of Fitz-Hugh-Curtis syndrome J Gynecol Obstet Biol Reprod (Paris) 1991 20 2 209 215 2071865

[B17] World Health Organisation (WHO) WHO report on the global tobacco epidemic, 2011 2011 Geneva: World Health Organisation

[B18] Lindow SW Moore PJ Ectopic pregnancy: analysis of 100 cases Int J Gynaecol Obstet 1988 27 3 371 375 10.1016/0020-7292(88)90115-4 2904900

[B19] Okonofua FE Ojo OS Odunsi OA Odesanmi WO Ectopic pregnancy associated with tubal schistosomiasis in a Nigerian woman Int J Gynaecol Obstet 1990 32 3 281 284 10.1016/0020-7292(90)90359-S 1972123

[B20] Bugalho A Strolego F Pregazzi R Osman N Ching C Extrauterine pregnancy in Mozambique Int J Gynaecol Obstet 1991 34 3 239 242 10.1016/0020-7292(91)90356-A 1673941

[B21] Ali AA Abdallah TM Siddig MF Diagnosis of ruptured ectopic pregnancy is still a challenge in Eastern Sudan Afr J Reprod Health 2012 15 4 106 108 22571112

[B22] Foumane P Mboudou ET Dohbit JS Ndingue SM Tebeu PM Doh AS Conservative treatment of ectopic pregnancy in a sub-Saharan African setting Trop Doct 2011 41 2 79 81 10.1258/td.2011.100085 21421884

[B23] Maouris P Review of 17 cases of ectopic pregnancy at the Vila central hospital in Vanuatu P N G Med J 1997 40 1 39 43 10365568

[B24] Ikeme AC Ezegwui HU Morbidity and mortality following tubal ectopic pregnancies in Enugu, Nigeria J Obstet Gynaecol 2005 25 6 596 598 10.1080/01443610500239552 16234149

[B25] Musa J Daru PH Mutihir JT Ujah IA Ectopic pregnancy in Jos Northern Nigeria: prevalence and impact on subsequent fertility Niger J Med 2009 18 1 35 38 19485145

[B26] Kouame N N’Goan-Domoua AM Meite A Konan AN Setcheou A Kone D N’Gbesso RD Keita AK Ultrasound and epidemiological features of ectopic pregnancy in a suburb of Abidjan (Cote d’Ivoire) Med Trop (Mars) 2012 71 5 481 483 22235622

[B27] Priuli G How I perform… salvage of peritoneal haemorrhage in ruptured ectopic pregnancies in Benin Gynecol Obstet Fertil 2008 36 4 455 456 10.1016/j.gyobfe.2008.01.013 18420443

[B28] Igberase GO Ebeigbe PN Igbekoyi OF Ajufoh BI Ectopic pregnancy: an 11-year review in a tertiary centre in the Niger Delta Trop Doct 2005 35 3 175 177 10.1258/0049475054620888 16105350

[B29] Priuli G Darate R Perrin RX Lankoande J Drouet N Multicentre experience with a simple blood salvage technique in patients with ruptured ectopic pregnancy in sub-Sahelian West Africa Vox Sang 2009 97 4 317 323 10.1111/j.1423-0410.2009.001215.x 19663935

[B30] Leke RJ Goyaux N Matsuda T Thonneau PF Ectopic pregnancy in Africa: a population-based study Obstet Gynecol 2004 103 4 692 697 10.1097/01.AOG.0000120146.48098.f2 15051561

[B31] Obed S Diagnosis of unruptured ectopic pregnancy is still uncommon in Ghana Ghana Med J 2006 40 1 3 7 17299556 PMC1790838

[B32] Thaddeus S Maine D Too far to walk: maternal mortality in context Soc Sci Med 1994 38 8 1091 1110 10.1016/0277-9536(94)90226-7 8042057

[B33] Kenneth G Population of PNG is more than 7 million 2012 Port Moresby: Post Courier http://www.postcourier.com.pg/20120404/news02.htm

[B34] United Nations Development Programme (UNDP) Papua New Guinea country profile: human development indicators http://hdrstats.undp.org/en/countries/profiles/PNG.html

[B35] Papua New Guinea National Statistics Office Papua New Guinea demographic and health survey of 2007 2009 Port Morseby: Papua New Guinea Government Printer

[B36] Hogan MC Foreman KJ Naghavi M Ahn SY Wang M Makela SM Lopez AD Lozano R Murray CJ Maternal mortality for 181 countries, 1980–2008: a systematic analysis of progress towards millennium development goal 5 Lancet 2010 375 9726 1609 1623 10.1016/S0140-6736(10)60518-1 20382417

[B37] World Health Organisation (WHO) Trends in maternal mortality: 1990–2010 2012 Geneva: World Health Organisation

[B38] Sanga K de Costa C Mola G A review of maternal deaths at goroka general hospital, Papua New Guinea 2005–2008 Aust N Z J Obstet Gynaecol 2010 50 1 21 24 10.1111/j.1479-828X.2009.01116.x 20218992

[B39] Sapuri M Klufio C A case of advanced viable extrauterine pregnancy P N G Med J 1997 40 1 44 47 10365569

[B40] Miller ED A 2000 gm tubal pregnancy Am J Obstet Gynecol 1987 156 5 1152 3578428 10.1016/0002-9378(87)90130-x

[B41] Mola G Aitken I Maternal mortality in Papua New Guinea 1976–1983 P N G Med J 1984 27 2 65 71 6336016

[B42] Mola G Maternal death in Papua New Guinea, 1984–1986 P N G Med J 1989 32 1 27 31 2750319

[B43] Alto W Is there a greater incidence of abdominal pregnancy in developing countries? Report of four cases Med J Aust 1989 151 7 412 414 2796817 10.5694/j.1326-5377.1989.tb101226.x

[B44] Ministry of Health of Papua New Guinea Manual of standard managements in obstetrics and gynaecology for doctors, H.E.O.s and nurses in Papua New Guinea 2010 6 Port Moresby: Ministry of Health

[B45] Amoko DH Buga GA Clinical presentation of ectopic pregnancy in Transkei, South Africa East Afr Med J 1995 72 12 770 773 8689974

[B46] Baffoe S Nkyekyer K Ectopic pregnancy in korle Bu teaching hospital, Ghana: a three-year review Trop Doct 1999 29 1 18 22 10418275 10.1177/004947559902900108

[B47] Gharoro EP Igbafe AA Ectopic pregnancy revisited in Benin City, Nigeria: analysis of 152 cases Acta Obstet Gynecol Scand 2002 81 12 1139 1143 10.1034/j.1600-0412.2002.811207.x 12519110

[B48] Bouyer J Coste J Shojaei T Pouly JL Fernandez H Gerbaud L Job-Spira N Risk factors for ectopic pregnancy: a comprehensive analysis based on a large case–control, population-based study in France Am J Epidemiol 2003 157 3 185 194 10.1093/aje/kwf190 12543617

[B49] Horne AW Brown JK Tong S Kaitu’u-Lino T Evaluation of ADAM-12 as a diagnostic biomarker of ectopic pregnancy in women with a pregnancy of unknown location PLoS One 2012 7 8 e41442 10.1371/journal.pone.0041442 22927907 PMC3424157

[B50] Wedderburn CJ Warner P Graham B Duncan WC Critchley HO Horne AW Economic evaluation of diagnosing and excluding ectopic pregnancy Hum Reprod 2009 25 2 328 333 19933287 10.1093/humrep/dep397 PMC2990466

[B51] Gare J Lupiwa T Suarkia DL Paniu MM Wahasoka A Nivia H Kono J Yeka W Reeder JC Mgone CS High prevalence of sexually transmitted infections among female sex workers in the eastern highlands province of Papua New Guinea: correlates and recommendations Sex Transm Dis 2005 32 8 466 473 10.1097/01.olq.0000161177.21639.96 16041247

[B52] Vallely A Page A Dias S Siba P Lupiwa T Law G Millan J Wilson DP Murray JM Toole M The prevalence of sexually transmitted infections in Papua New Guinea: a systematic review and meta-analysis PLoS One 2010 5 12 e15586 10.1371/journal.pone.0015586 21203468 PMC3009733

[B53] UNAIDS Country progress report: Papua New Guinea 2012 Port Moresby: UNAIDS

[B54] Kasule J Seeras R Ectopic tubal pregnancy in Zimbabwe J Obstet Gynaecol (Lahore) 1989 9 3 180 183 10.3109/01443618909151030 12282923

[B55] Kouam L Kamdom-Moyo J Doh AS Ngassa P Treatment of ectopic pregnancies by laparotomy in under-equipped countries. A series of 144 cases at the yaounde university hospital center (Cameroon) J Gynecol Obstet Biol Reprod (Paris) 1996 25 8 804 808 9026508

[B56] Pal A Gupta KB Sarin R A study of ectopic pregnancy and high risk factors in Himachal Pradesh J Indian Med Assoc 1996 94 5 172 173 202 8855569

[B57] Senn M Baiwog F Winmai J Mueller I Rogerson S Senn N Betel nut chewing during pregnancy, Madang province, Papua New Guinea Drug Alcohol Depend 2009 105 1–2 126 131 19665325 10.1016/j.drugalcdep.2009.06.021

[B58] Burnet Institute Policy report: improving maternal, newborn and child health in Papua New Guinea through family and commuity health care 2012 Melbourne: Burnet Institute

